# Temperature Elevation Alters the Gut Antibiotic Resistome and Carbohydrate-Active Enzymes in the Desert Lizard *Eremias roborowskii*

**DOI:** 10.3390/microorganisms14051084

**Published:** 2026-05-11

**Authors:** Yuhan Zheng, Ruichen Wu, Huawei Feng, Xunheng Wu, Yi Yang

**Affiliations:** 1 Xinjiang Key Laboratory for Ecological Adaptation and Evolution of Extreme Environment Organisms, College of Life Sciences, Xinjiang Agricultural University, Urumqi 830052, China; zyhzyh_yuhanzheng@163.com (Y.Z.); wuwuwurc@163.com (R.W.); 17554223065@163.com (H.F.); 19861765873@163.com (X.W.); 2 Xinjiang Agricultural University Wildlife Gut Microecology and Metabolism Research Center, College of Life Sciences, Xinjiang Agricultural University, Urumqi 830052, China

**Keywords:** climate warming, heat stress, *Eremias roborowskii*, antibiotic resistance genes (ARGs), carbohydrate-active enzymes (CAZymes)

## Abstract

In the context of global warming, the resulting persistent thermal stress has become a critical environmental factor influencing the structural and functional homeostasis of gut microbiota in reptiles. In this study, *Eremias roborowskii*, a desert lizard endemic to the extreme heat conditions of the Turpan Basin, was selected as an ideal model for evaluating the ecological impacts of global warming. Meanwhile, a 60-day controlled laboratory experiment was conducted, exposing the lizards to normal (30 °C ± 1 °C), elevated (37 °C ± 1 °C), and high (42 °C ± 1 °C) temperatures to reflect future climate scenarios. Using shotgun metagenomic sequencing, the gut microbiota was characterized to investigate the dynamics of the antibiotic resistance genes (ARGs) and carbohydrate-active enzymes (CAZymes) under heat stress. The results reveal that elevated temperature selectively promotes heat-tolerant gut microbiota, such as *Tetragenococcus* and *Faecalicatena*, by altering host energy metabolism and modulating heat stress adaptation to maintain intestinal homeostasis. Moreover, the observed increase in resistome diversity and richness under elevated temperature may be attributed to temperature-induced shifts in gut microbial composition, particularly the enrichment of heat-tolerant ARG-carrying bacterial taxa. Metabolic changes in CAZymes were caused by gut microbiota remodeling, which optimized carbon utilization and preferentially allocated cell wall synthesis and repair. Furthermore, the pentose phosphate pathway and amino acid biosynthesis pathways were upregulated, providing NADPH for antioxidant defense and precursors for protein synthesis, respectively, thereby contributing to the maintenance of microbial cellular homeostasis. Our study provides a theoretical basis for understanding functional gene adaptation strategies in wildlife microbiomes due to climate change.

## 1. Introduction

Climate warming has become a worldwide threat to the health of mankind and the biosphere due to anthropogenic greenhouse gas emissions and land use changes [1]. According to the Sixth Assessment Report (AR6) from the Intergovernmental Panel on Climate Change (IPCC), the global surface temperature was 1.09 °C higher in 2011–2020 than in 1850–1900, with larger increases over land (1.59 °C) than over the ocean (0.88 °C) [2]. Consequently, land areas have experienced a persistent warming trend, which has become more serious and is accompanied by an increasing frequency of extreme heat events. Among them, heatwave events are characterized by a continuous process of abnormally high temperatures [3], often occurring at rates and intensities that exceed the adaptive capacity of organisms. Ultimately, such persistent heat extremes not only deteriorate the health status of wildlife, weaken environmental resilience [4], and increase the likelihood of the spread of dangerous diseases [5,6], but also accelerate the extinction rate and biodiversity loss of populations [7].

Antibiotic resistance genes (ARGs) are a major concern for universal public health [8], and antibiotics act as selective stressors that induce environmental microorganisms to produce and enrich ARGs, thereby posing a potential threat to ecosystem stability. However, antibiotic resistance genes are also naturally present in microbial communities regardless of human influence, which may be a side effect of the original function of certain gene products, such as efflux pumps [9]. In particular, temperature exerts a significant impact on the emergence, distribution, and dissemination of ARGs [10]. Studies have demonstrated that when the water temperature of the Yellow River rises from 23 to 35 °C, the diversity of ARGs decreases significantly while their abundance increases [11]. It has also been reported that elevated temperatures can lead to a significant increase in the abundance of ARGs in the gut of soil collembolans [12]. Furthermore, high-temperature stress drives dynamic changes in the ecological niches of microbial communities by regulating the proliferation and competition of host bacteria, thereby influencing the host range, carrying abundance, environmental fate, and transmission risk of ARGs [13].

The gut microbiome, which is composed of the phyla Firmicutes and Bacteroidetes, has been shown to play a crucial role in maintaining host health and is involved in the digestion, metabolism, immunity, and defense against pathogens [14,15]. The composition of animal gut microbiota has been demonstrated to be influenced by numerous factors, including temperature, diet, season, and pathogen exposure [16]. Notably, gut microbiota dedicate substantial percentages of their genomes to the degradation and uptake of carbohydrates, indicating the importance of this class of molecules, namely carbohydrate-active enzymes (CAZymes). Carbohydrates function not only as a carbon source for these bacteria but also as a means of attachment to the host and a barrier to infection of the host [17]. Meanwhile, these enzymes have been shown to be crucial for the degradation, modification, and synthesis of carbohydrates, facilitating the breakdown of complex dietary polysaccharides [18]. This process has been demonstrated to compensate for the host’s metabolic limitations while enabling the utilization of recalcitrant carbon sources [19]. Understanding the diversity and function of CAZymes in the gut microbiota can reveal the mechanisms of species’ adaptation to the survival environment, especially in regions where food sources may be limited and require specialized digestive capabilities [20].

*Eremias roborowskii* (Bedriaga, “1905”, 1907) (genus *Eremias*, family Lacertidae) is a diurnal endemic species in the Turpan Basin in the Xinjiang Uyghur Autonomous Region of China [21], which is one of the most arid and water-insecure regions in China [22]. Since 1980, climate change has left marks on the ecosystem in Northwestern China, including this area, with steadily rising temperatures [23]. In particular, *E. roborowskii* feeds primarily on insect-based food and only consumes the mature fruits of *Capparis spinosa* (L., 1753) seasonally (in summer) [24]. It is likely driven by the severely limited food availability that results from the combined effects of climate and extreme environmental conditions, necessitating a supplemental intake of water and carbon sources for survival. Studies have shown that the sympatric lizard *Teratoscincus roborowskii* also exhibits seasonal dietary shifts, during which the gut microbiota is reshaped to enrich microbes involved in plant nutrient metabolism [24,25,26]. Meanwhile, ARGs and CAZymes are critical functional components of the gut microbiota, respectively playing pivotal roles in mediating microbial environmental adaptation and driving host nutritional metabolism. Therefore, we hypothesized that climate-induced heat stress may alter host physiology, reshaping the gut microbial community, thereby modulating both the antibiotic resistome and carbohydrate metabolism capacity, representing a complementary mechanism of microbial adaptation to extreme environments. In this study, metagenomic sequencing was employed to characterize the composition and variation in ARGs and CAZymes in the gut microbiome of *E. roborowskii* under laboratory-controlled heat stress. Our study will provide a theoretical basis for evaluating the potential risks of ARGs and CAZymes to the health of wild *E. roborowskii* under global climate change and contribute to understanding the reshaping of host microbiomes under extreme environmental conditions.

## 2. Materials and Methods

### 2.1. Test Animals

The 19 male *E*. *roborowskii* were all adult and captured in August 2024 in the Turpan region, which is located in the Turpan Basin in the Xinjiang Uyghur Autonomous Region of China (42°51′40.621′′ N and 88°52′1.082′′ E), at an altitude of −62 m. All experimental procedures were approved by the Animal Welfare and Ethics Committee of Xinjiang Agricultural University, Urumqi, Xinjiang, China (approval number: 2023031).

### 2.2. Feeding Environment and Temperature Treatment

All lizards were housed in 520 mm × 355 mm × 285 mm (L × W × H) rearing boxes covered with soil and sand substrates to simulate their natural habitats with sufficient shelter, and they were provided with sunlamps (UVB + UVA 3.0, 50 W) for 12 h (08:30–20:30) per day for ambient temperature taming. During the period of domestication, the lizards were provided with sufficient food, including mealworms and water, and nutrients such as calcium powder and vitamins to maintain the nutritional requirements of *E*. *roborowskii* [27].

After a period of acclimation and domestication, *E. roborowskii* individuals were randomly assigned to different temperature groups for a 60 d temperature-controlled experiment. According to the projections for the most severe greenhouse gas emission scenario (SSP5-8.5) established in the IPCC AR6, the global average temperature is very likely to rise from 3.3 to 5.7 °C by 2081–2100 [2]. Therefore, three temperature treatment groups were set up: the normal-temperature group (NT, *n* = 6) at 30 °C ± 1 °C, the elevated-temperature group (ET, *n* = 6) at 37 °C ± 1 °C, and the high-temperature group (HT, *n* = 7) at 42 °C ± 1 °C. The temperature-controlled simulation experiment was conducted under a natural circadian rhythm, with 12 h of illumination (8:30–20:30) provided by sunlamps (UVB + UVA 3.0, 100 W) to strictly control the target temperatures for each treatment, while eliminating variations in light intensity. All other captive conditions were kept consistent with those used in the NT group. A thermocouple temperature recorder (Source-Hengtong YHT309S, range: −200 °C to 1370 °C; resolution: 0.01 °C) was used to monitor the rearing environment temperature of the *E*. *roborowskii*.

**Figure 1:**
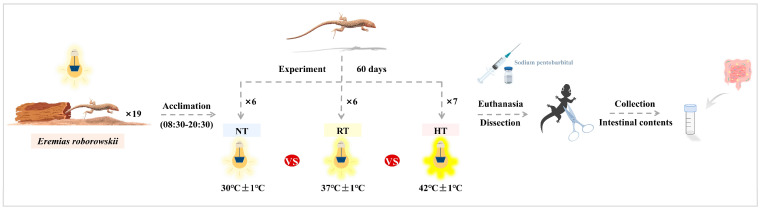
The experimental design of the temperature treatment.

The *E. roborowskii* in the three groups was first euthanized with sodium pentobarbital [28,29]. The external part of the cloaca and the dissection tools were thoroughly cleaned with ethanol to reduce the interference of environmental microbiota. Subsequently, the abdominal cavity of the lizard was opened, and its intestine was removed. Intestinal contents from the large intestine were collected and placed into a sterile cryotube. The sterile cryotubes were temporarily stored in a liquid nitrogen tank. All samples were subsequently stored at −80 °C [30] (Figure 1).

### 2.3. Metagenomic Sequencing

Nucleic acids are extracted using the TGuide S96 Magnetic Universal DNA Kit. The extracted nucleic acids are quantified with NanoDrop2000 (Thermo Fisher, Waltham, MA, USA) and Qubit™3 Fluorometer (Thermo Fisher, Waltham, MA, USA), and their integrity was assessed via agarose gel electrophoresis (Electrophoresis System: Tanon, EPS60; Electrophoresis Tank: Tanon, EPS600, Shanghai Tanon Life Science Co., Ltd., Shanghai, China). Library construction is performed using the VAHTS^TM^ Universal Plus DNA Library Prep Kit for Illumina (ND617-02, BiOptic Inc., Taipei, Taiwan, China), involving steps like DNA fragmentation (enzymatic digestion) and end repair, adapter ligation, fragment size screening, and PCR amplification, with products purified using VAHTSTM DNA Clean Beads (N411-03, Thermo Fisher, Waltham, MA, USA). Library quality control is conducted with Qsep-400 for fragment inspection and Qubit 3.0 for concentration quantification, meeting criteria such as concentration ≥1 ng/μL, specific fragment size, normal peak shape, and a single fragment without contaminants. The qualified libraries are sequenced on the Illumina NovaSeq X platform with a paired-end (PE) 150 strategy. The raw reads obtained from sequencing are subjected to quality control and filtering to generate clean reads, which are used for subsequent bioinformatics analysis.

### 2.4. Data Analysis

Raw Tags were filtered using Fastp software(v0.23.1) to obtain high-quality sequencing data [31]. The base distribution and quality of the clean reads under different temperature treatments are shown in Supplementary Figure S1 and Supplementary Figure S2, respectively. Prior to the library construction, target DNA fragments were >2000 bp, and the library insert size exhibited a main peak of 400–600 bp. Sequencing was performed on the Illumina NovaSeq X platform (PE150). Two 150 bp reads were generated from each end of the insert, covering the terminal regions without overlapping in the central region. The primary alignment strategy was local alignment, supplemented by global alignment. Repetitive reads were filtered out, and multi-mapping reads were removed. For indels, appropriate gap penalties were set; the central unsequenced gap region was defined as such, with no base filling or assembly performed. Host genome contamination was removed by aligning with the host genome sequence using Bowtie2 (v2.2.4) [32]. Metagenomic assembly was performed using MEGAHIT software (v1.1.2), and contigs shorter than 300 bp were filtered out [33]. Meanwhile, the 300 bp cutoff refers to the maximum sequencing read length per single read, rather than the insert size. The effective alignment length per single end was ≥150 bp, and the combined length of both ends was ≥300 bp. The assembly results are evaluated using QUAST software (v2.3) [34]. Coding regions in the genome are identified using MetaGeneMark software (http://exon.gatech.edu/meta_gmhmmp.cgi, v3.26, accessed on 8 May 2025) with the default parameters [35]. Redundancy is removed using MMseqs2 software (https://github.com/soedinglab/mmseqs2, v11-e1a1c, accessed on 1 June 2025) with a similarity threshold of 90% and a coverage threshold of 80% [36]. The non-redundant gene set is then functionally annotated using the Comprehensive Antibiotic Resistance Database (CARD) and Carbohydrate-Active Enzymes Database (CAZy), along with taxonomic analysis to calculate the species composition and abundance information of the samples.

Alpha diversity, beta diversity, and inter-group significant difference analyses were performed using the BMKCloud platform (http://www.bio-cloud.net/, accessed on 22 May 2025); one-way analysis of variance (one-way ANOVA) was applied for the inter-group significant difference analysis (*p* < 0.05). One-way ANOVA and a Benjamini–Hochberg false discovery rate correction (BH-FDR) for multiple testing were used to identify differentially abundant gut microbial taxa, ARGs, and CAZymes. These significantly different features were subsequently visualized and interpreted through heatmap clustering analysis. For the ARGs and CAZymes across the three temperature treatment groups, correlation network analyses were performed using the Spearman correlation method with a correlation coefficient threshold of 0.5 and a *p*-value threshold of 0.05. Meanwhile, GraphPad Prism 10.1.2 software and the ChiPlot online tool (https://www.chiplot.online/, accessed on 28 May 2025) were used for graphing. In addition, correlation analyses between differential gut microbial taxa and differential ARGs, between differential gut microbial taxa and differential CAZymes, and between differential CAZymes and differential Kyoto Encyclopedia of Genes and Genomes (KEGG) pathways were all conducted using the linkET package in R software (v4.4.1).

## 3. Results

### 3.1. Analysis of Gut Microbiota Diversity and Differential Abundance in E. roborowskii Under Different Temperature Treatment

**Figure 2:**
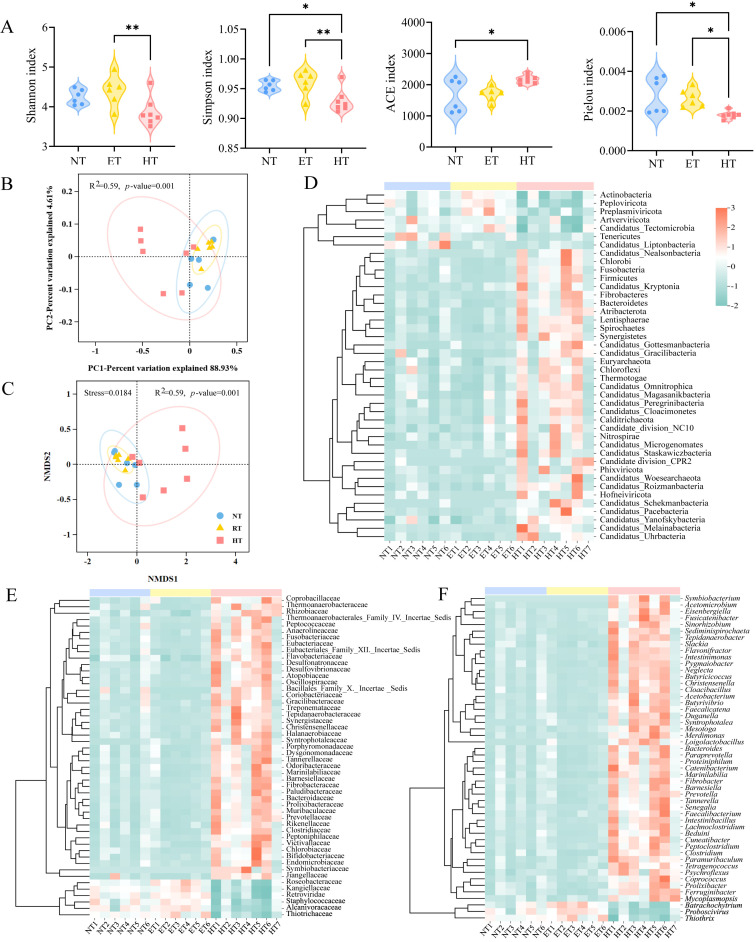
Gut microbiota diversity and the heatmap of differential abundance in *E. roborowskii* under different temperature treatments. Alpha diversity of gut microbiota among three groups at the genus level (**A**). PCoA (**B**) and NMDS (**C**) analysis of ARGs among three groups at the genus level. The heatmap of differential abundance at the phylum (top 42) (**D**), family (top 50) (**E**), and genus (top 50) (**F**) levels. * *p* < 0.05, and ** *p* < 0.01.

Significant differences in alpha diversity were observed at the genus level, with the diversity and richness slightly higher in the HT group compared to the NT group (Figure 2A). The PCoA and NMDS plots revealed a separation among the three groups and that there was a significant difference between them (Figure 2B,C). Moreover, the distribution of the microbial community was more discrete in the HT group, while it appeared more uniform in the NT group. The heatmap of differential gut microbiota abundance under different temperature treatments in *E. roborowskii* can be seen in Figure 2. The results indicated that at the phylum level, the relative abundances of Firmicutes, Bacteroidetes, and Fibrobacteres were significantly higher, while Peploviricota was significantly lower in the HT group compared to the NT and ET groups (Figure 2D). Furthermore, the NT group exhibited a significantly higher relative abundance of Candidatus Liptonbacteria compared to both the ET and HT groups. At the family level (Figure 2E), the relative abundances of the Rhizobiaceae, Prevotellaceae, and Eubacteriales_Family_XII._Incertae_Sedis were significantly higher in the HT group than in the NT and ET groups, whereas the abundances of Alcanivoracaceae, Thiotrichaceae, and Kangiellaceae were significantly lower. At the genus level (Figure 2F), the relative abundances of *Tetragenococcus*, *Faecalicatena*, and *Merdimonas* were significantly higher in the HT group compared to the NT and ET groups, while the relative abundances of *Proboscivirus* and *Thiothrix* were significantly lower than those observed in the other two groups.

### 3.2. Analysis of ARGs Diversity of Gut Microbiota in E. roborowskii Under Different Temperature Treatments

**Figure 3:**
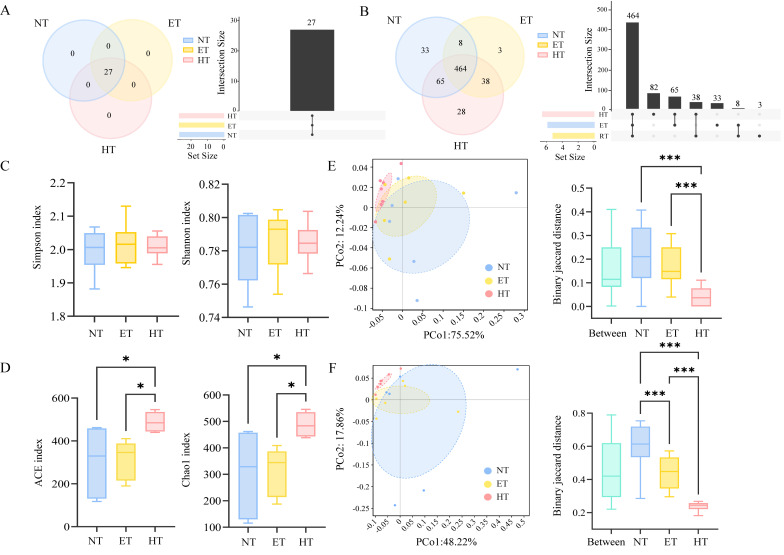
ARGs’ diversity of gut microbiota in *E. roborowskii* under different temperature treatments. A Venn and UpSet diagram of ARGs among three groups at the resistance-formatted (**A**) and ARO (**B**) levels; alpha diversity of ARGs among three groups at the resistance-formatted (**C**) and ARO (**D**) levels; and PCoA and PERMANOVA analysis of ARGs among three groups at the resistance-formatted (**E**) and ARO (**F**) levels. * *p* < 0.05, and *** *p* < 0.001.

Based on CARD annotation, the diversity of ARGs was analyzed. At the resistance-formatted level, a total of 27 ARGs were identified, and all of them were shared among the three groups (Figure 3A). At the antibiotic resistance ontology (ARO) level, a total of 639 ARGs were identified (Figure 3B). The HT group exhibited the highest number of ARGs (595), with 28 being unique, followed by the NT (570; 33 unique) and RT groups (513; 3 being unique). No significant differences were observed among the three temperature treatments in the alpha diversity indices of ARGs at the resistance-formatted level (Figure 3C). In contrast, at the ARO level, the ACE and Chao1 indices were significantly higher in the NT and RT groups than in the HT group (Figure 3E). The PCoA results indicated a significant separation between the NT and HT groups at both the resistance-formatted and ARO levels. PERMANOVA further confirmed no significant differences at the resistance-formatted level (R^2^ = 0.172, *p* = 0.195), whereas a significant difference was detected among the three groups at the ARO level (R^2^ = 0.255, *p* = 0.012).

### 3.3. Composition, Differences, and Correlation Network of ARGs of Gut Microbiota in E. roborowskii Under Different Temperature Treatments

**Figure 4:**
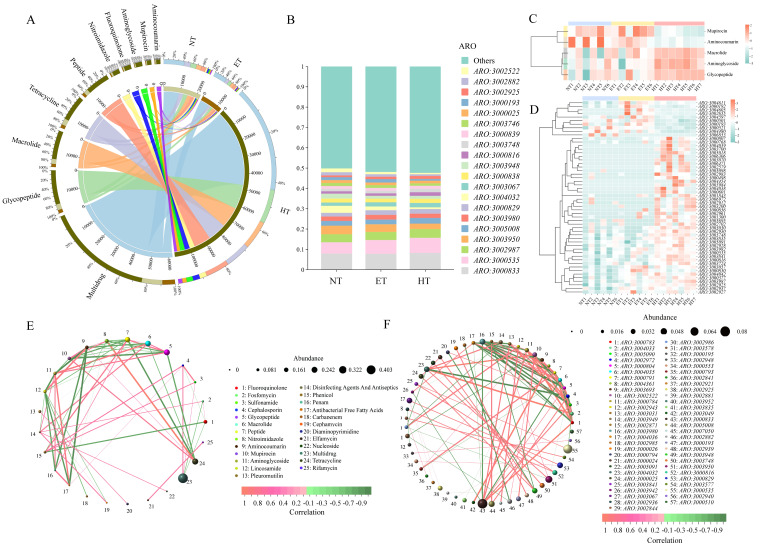
Composition, differences, and correlation networks of the ARGs of gut microbiota in *E. roborowskii* under different temperature treatments. Relative abundance of ARGs among three groups at the resistance-formatted (**A**) and ARO (**B**) levels. Differential analysis of ARGs among three groups at the resistance-formatted (**C**) and ARO (**D**) levels. Correlation network analysis of ARGs among three groups at the resistance-formatted (**E**) and ARO (**F**) levels.

The abundances of ARGs under the different taxonomic classifications can be found in Figure 4. At the resistance-formatted level, the ARGs in the NT, ET, and HT groups were dominated by multidrug (39.90%, 40.19%, and 40.66%), tetracycline (14.77%, 12.54%, and 9.89%), and glycopeptide (9.87%, 10.96%, and 12.96%), while other representative ARGs included macrolides and peptides (Figure 4A). At the ARO level, the ARGs in the NT group were dominated by *ARO:3000833*, *ARO:3000535*, and *ARO:3003950*. Compared with the NT group, the dominant ARGs in the ET and HT groups included *ARO:3000833*, *ARO:3000535*, and *ARO:3002987* (Figure 4B).

Furthermore, the differential abundance of ARGs of gut microbiota in *E. roborowskii* under different temperature (30 °C, 37 °C, and 42 °C) treatments was analyzed. At the resistance-formatted level, the relative abundances of mupirocin and aminoglycoside in the HT group were significantly lower than those in the other two groups, whereas the relative abundances of aminocoumarin, macrolide, and glycopeptide in the HT group were significantly higher than in the NT group (Figure 4C). At the ARO level, in comparison with the HT group, the relative abundances of *ARO:3000816*, *ARO:3003841*, and *ARO:3003030* were significantly higher than in the NT and ET groups, while *ARO:3000501* was significantly less abundant in HT (Figure 4D). In the ET and HT groups, the relative abundances of *ARO:3004042*, *ARO:3003057*, and *ARO:3002937* were significantly elevated relative to the NT group, whereas the abundance of *ARO:3006853* was significantly reduced.

Correlation network analysis of ARGs was conducted across the three temperature treatments, with increasing temperature, and strong positive correlations at the resistance-formatted level were observed between nitroimidazole and tetracycline (r = 0.81), lincosamide and aminocoumarin (r = 0.78), and aminoglycoside and macrolide (r = 0.75). Conversely, highly negative correlations were observed between aminoglycoside and aminocoumarin (r = −0.89), antibacterial free fatty acids and aminocoumarin (r = −0.82), and mupirocin and glycopeptide (r = −0.78) (Figure 4E). At the ARO level, strong positive correlations were detected between *ARO:3000783* and *ARO:3000784* (r = 0.95), between *ARO:3004033* and *ARO:3004032* (r = 0.91), and between *ARO:3002522* and *ARO:3004032* (r = 0.86), whereas a strong negative association was found between *ARO:3002972* and *ARO:3004032* (r = −0.89), between *ARO:3002972* and *ARO:3004033* (r = −0.88), and between *ARO:3004035* and *ARO:3000784* (r = −0.88) (Figure 4F).

### 3.4. Analysis of CAZymes Diversity of Gut Microbiota in E. roborowskii Under Different Temperature Treatments

**Figure 5:**
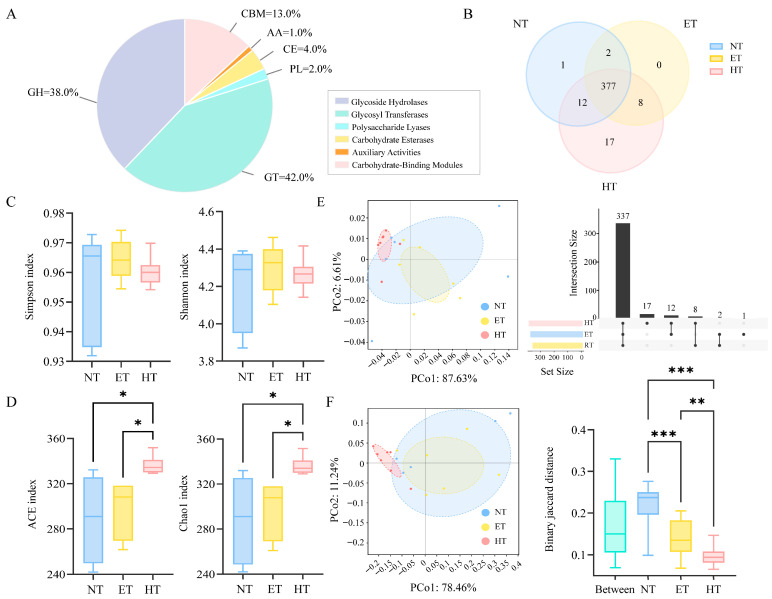
CAZymes diversity of gut microbiota in *E. roborowskii* under different temperature treatments. (**A**) The composition of CAZymes at the class level. (**B**) A Venn and UpSet diagram of CAZymes at the family level. Alpha diversity of CAZymes among three groups at the class (**C**) and family (**D**) levels. PCoA and PERMANOVA analysis of CAZymes among three groups at the class (**E**) and family (**F**) levels. * *p* < 0.05, ** *p* < 0.01, and *** *p* < 0.001.

Functional genes were aligned against the CAZy database, and at the class level, a total of six CAZymes were identified (Figure 5A). Glycosyl transferases (GT) were the most abundant, followed by glycoside hydrolases (GH), carbohydrate-binding modules (CBM), carbohydrate esterases (CE), polysaccharide lyases (PL), and auxiliary activities (AA). As shown in Figure 5B, at the family level, 417 CAZymes were identified across the three treatment groups. There were 414 CAZymes in the HT group, including 17 unique families; the NT group contained 392 CAZymes, with only one unique family; and the ET group had 387 CAZyme families with none that were unique. CAZymes alpha diversity indices at the class level revealed no significant differences among the three temperature treatments (Figure 5C). In contrast, at the family level, ACE and Chao1 indices were significantly higher in the HT group than in the other two groups, indicating higher alpha diversity (Figure 5E). PCoA showed no significant separation at the class level, whereas significant separation was observed at the family level. PERMANOVA analysis further confirmed the significant differences at the family level (R^2^ = 0.317, *p* = 0.003).

### 3.5. Composition, Differential Abundance, and Correlation Network of CAZymes of Gut Microbiota in E. roborowskii Under Different Temperature Treatments

**Figure 6:**
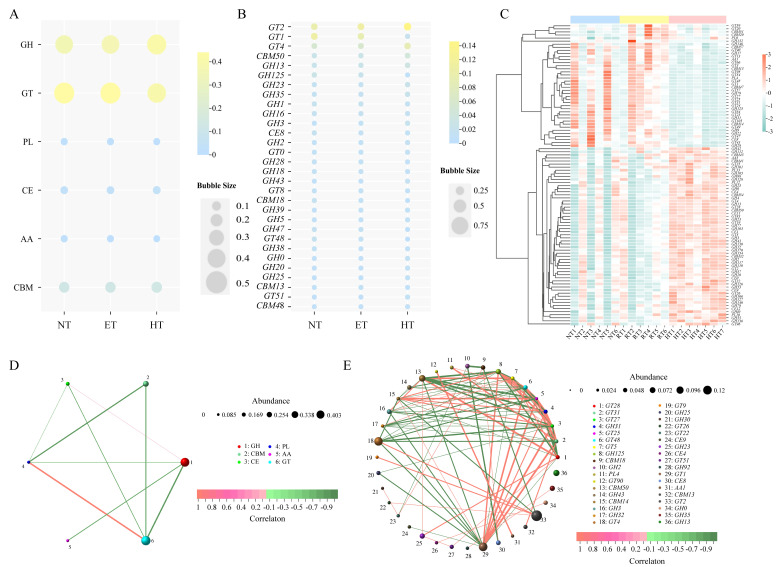
Composition, differences, and correlation networks of the CAZymes of gut microbiota in *E. roborowskii* under different temperature treatments. Relative abundance of CAZymes among three groups at the class (**A**) and family (**B**) levels. (**C**) Differential analysis of CAZymes among three groups at the family level. Correlation network analysis of CAZymes among three groups at the class (**D**) and family (**E**) levels.

The abundances of CAZymes at the class (enzyme functional category) and family (enzyme family) levels are shown in Figure 6. At the class level, the CAZymes in the NT, ET and HT were mainly dominated by GH (36.27%, 36.51%, and 41.08%), GT (36.27%, 44.02%, and 39.32%), and CBM (12.55%, 12.72%, and 13.20%), whereas other representative classes included CE (3.56%, 3.39%, and 4.10%), PL (1.73%, 1.73%, and 1.38%) and AA (1.81%, 1.63%, and 0.91%) (Figure 6A). At the family level, the CAZymes in the NT and ET groups were dominated by *GT1*, *GT2*, and *GT4*, while in the HT group, the dominant CAZymes included *GT2*, *GT4*, and *CBM50* (Figure 6B). At the family level, in comparison with the HT group, the relative abundances of *CBM74*, *GH53*, and *GH3* were significantly higher than those of the NT and ET groups, while *PL3*, *PL4*, and *GH31* were significantly less abundant in the HT group. In the ET and HT groups, the relative abundances of *GH28*, *GT49*, and *GT105* were significantly reduced relative to those of the NT group (Figure 6C).

Correlation network analysis results revealed that, with increasing temperature, strong positive correlations at the class level were identified between PL and GT (r = 0.90) and between CE and GH (r = 0.57). Conversely, there is a highly negative correlation between GH and GT (r = −0.78), PL and CBM (r = −0.73), and CBM and GT (r = −0.70) (Figure 6D). At the family level, a strong positive association was detected between *GT28* and *CBM50* (r = 0.99), *GT31* and *GT1* (r = 0.99), and *GT48* and *GT1* (r = 0.98). Meanwhile, strong negative correlations were found between *GH31* and *CBM50* (r = −0.99), *GT28* and *GH31* (r = −0.99), and *GH125* and *CBM50* (r = −0.98) (Figure 6E).

**Figure 7:**
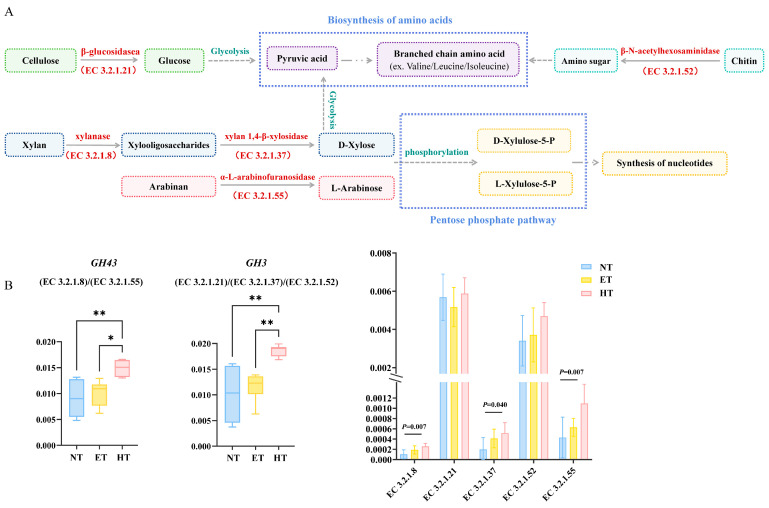
Elevated temperature alters gut microbial KEGG pathways functionally associated with CAZymes in *E. roborowskii*. (**A**) The pentose phosphate pathway (ko00030) and Biosynthesis of amino acids (ko01230); and (**B**) the relative abundance of CAZymes and key enzymes. * *p* < 0.05, and ** *p* < 0.01.

In the HT group, the enrichment of the *GH43* family led to the upregulation of its regulated enzymes, xylanase (EC 3.2.1.8) and arabinanase (EC 3.2.1.55) (Figure 7A). These enzymes degrade hemicellulose to xylose and arabinose, which are subsequently phosphorylated and converted to key intermediates of the pentose phosphate pathway (PPP), L-xylulose-5-phosphate (L-Xylulose-5-P), and D-xylulose-5-phosphate (D-Xylulose-5-P), thereby feeding into the pathway and providing nucleotide synthesis and reducing power (NADPH) to maintain redox homeostasis. Concurrently, β-glucosidase (EC 3.2.1.21) and endo-1,3-1,4-β-glucanase (EC 3.2.1.37), regulated by the *GH3* family, participate in cellulose degradation, yielding glucose (Figure 7B). Glucose is further converted via glycolysis to pyruvate, which serves as a central carbon skeleton providing precursors for the biosynthesis of amino acids. The relative abundance of these CAZymes was significantly higher in the HT group than in the NT and ET groups. In addition, chitin is decomposed into amino sugars by *GH3*-encoded enzymes (EC 3.2.1.52), which then participate in the amino acid biosynthesis pathway.

### 3.6. Correlation Analysis of Gut Microbiota, ARGs, CAZymes, and KEGG Pathways in E. roborowskii Under Different Temperature Treatment

**Figure 8:**
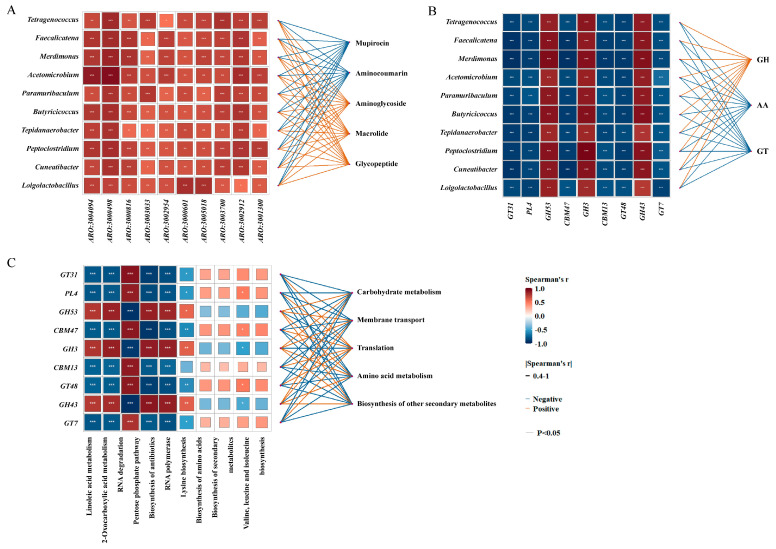
The correlation analysis of different gut microbiota, ARGs, CAZymes, and KEGG pathways. (**A**) A correlation heatmap of different ARGs at the resistance-formatted (top 5) and ARO (top 10) levels, and different gut microbiota (top 10) at the genus level; (**B**) a correlation heatmap of different CAZymes at the class (top 3) and family (top 10) levels, and different gut microbiota (top 10) at the genus level; and (**C**) a correlation heatmap of different KEGG pathways at the secondary (top 5) and tertiary (top 10) levels, and different CAZymes. * *p* < 0.05, ** *p* < 0.01, and *** *p* < 0.001.

Correlation analysis between the differentially abundant gut microbiota and ARGs revealed varying degrees of positive and negative associations (Figure 8A). At the resistance-formatted level, significant positive correlations were observed between aminoglycoside, macrolide, and glycopeptide resistance genes and the differential microbiota. In contrast, mupirocin and aminocoumarin resistance genes exhibited significant negative correlations. At the ARO level, all differential ARGs showed significant positive correlations with the differential microbiota. A correlation heatmap was constructed to evaluate the relationships between the differential gut microbiota and CAZymes (Figure 8B). At the class level, GH were significantly positively correlated with the differential microbiota, while AA and GT showed significant negative correlations. At the family level, *GH53*, *GH3*, and *GH43* exhibited significant positive correlations, while *GT31*, *PL4*, and *CBM47* were significantly negatively correlated with the differential microbiota. A correlation heatmap was generated to examine the associations between the differential CAZymes and KEGG pathways (Figure 8C). At the secondary level, carbohydrate metabolism, membrane transport, and amino acid metabolism were significantly positively correlated with *GH53*, *GH3*, and *GH43*, but significantly negatively correlated with *GT31*, *PL4*, and *CBM47*. Whereas, translation was significantly positively correlated with *GT31*, *PL4*, and *CBM13*, and significantly negatively correlated with *GH53*, *GH3*, and *GH43*. At the tertiary level, linoleic acid metabolism, 2-oxocarboxylic acid metabolism, and the PPP were significantly positively correlated with *GH53*, *GH3*, and *GH43*, and significantly negatively correlated with *GT31*, *PL4*, and *CBM47*. In contrast, RNA degradation and the biosynthesis of secondary metabolites showed the opposite pattern.

## 4. Discussion

### 4.1. Elevated Temperature Reshapes the Diversity of ARGs of Gut Microbiota in E. roborowskii

The profiles of ARGs were significantly altered by temperature, with ARG diversity decreasing markedly as temperature increased. Similar pathways can be co-opted by organisms to respond to multiple stressors. Additionally, temperature-induced stress responses have been co-opted to deal with antibiotic stress [37]. Furthermore, high-temperature stress may potentially promote horizontal gene transfer (HGT) by enhancing its frequency through elevated reactive oxygen species (ROS) and ATP levels [10], as well as by improving the transfer efficiency of mobile genetic elements (MGEs), thereby accelerating the dissemination of ARGs [38]. This phenomenon may drive thermal adaptation in microorganisms and concomitantly affect antibiotic resistance levels. Notably, in our study, significant differences in gut microbiota diversity were observed, which may reflect the microbial survival under thermal stress and indicate that elevated temperature reshaped the gut microbial community by eliminating heat-sensitive taxa and enriching heat-tolerant taxa. Consequently, shifts in antibiotic resistance largely stemmed from these community changes. These mechanisms may explain the highest number of ARGs observed at the ARO level in the HT group, which is significantly higher than the NT and ET groups. Furthermore, with increasing temperature, the beta diversity of ARGs exhibits a convergent trend, which reflects reduced structural variability and enhanced community homogeneity. Although PCoA did not reveal definite separation among the three groups, PERMANOVA confirmed significant differences in ARGs composition at the ARO level under different temperature treatments, which supports the conclusion that the ARG community structure becomes more homogenized under thermal stress.

Numerous studies have reported that microbial communities are shaped by deterministic factors such as environmental selection and bio-interactions, and stochastic processes such as speciation, birth, death, and dispersal limitation [37,38,39,40,41]. These theories have also been used to explore the assembly of ARG communities [42]. It was shown that the abundance of glycopeptide resistance genes was significantly higher in the gut microbiota of *Macacamulatta vestitas* (Milne-Edwards, 1892) at high altitudes compared to *Semnopithecus schistaceus* (Hodgson, 1840) at mid–low altitudes, suggesting that extreme environments may drive the selective enrichment of specific antibiotic resistance genes [20]. Furthermore, a high temperature can alter membrane permeability, denature protein structures, and inactivate metabolic enzymes, preventing the survival of temperature-sensitive microorganisms [42,43]. However, elevated temperatures also promote the enrichment of microbiota with thermal tolerance and the capacity to carry multiple ARGs. Thus, elevated temperatures may selectively eliminate thermally sensitive microbiota taxa, and thermally tolerant microbiota gain a competitive advantage, which may explain why the relative abundance of resistance genes, including multidrug, tetracycline, and glycopeptide, exhibited significant differences with increasing temperature in our study. Additionally, at the ARO level, we found that the NT group was dominated by *ARO:3000833*, *ARO:3000535*, and *ARO:3003950*, whereas the ET and HT groups were characterized by *ARO:3000833*, *ARO:3000535*, and *ARO:3002987*, indicating a temperature-mediated selection of specific microbiota hosts. The increase in *ARO:3002987* at a high temperature is likely associated with the proliferation of thermotolerant microbiota taxa. Furthermore, *ARO:3000816*, *ARO:3003841*, and *ARO:3003030* were significantly enriched in the HT group, which is consistent with a previous study where elevated temperature remarkably increased the abundance of ARGs in the gut microbiota of *E. roborowskii* [44].

A high temperature may reshape the distribution and associations of ARGs through dual mechanisms. On the one hand, it enhances the transfer efficiency of MGEs, thereby facilitating the co-selection of resistance genes located on the same genetic element. ARGs can not only be stably inherited between microbial generations via vertical gene transfer but also horizontally transferred between different microbial species with the help of MGEs such as plasmids, transposons, and integrons, accelerating the spread and diffusion of resistance genes in microbial communities [45,46]. Furthermore, this process corresponds to the gene co-localization hypothesis, which indicates that multiple genes, including those encoding different resistance phenotypes, tend to exhibit synchronized genetic transfer, functional expression, and regulatory coordination due to their physical linkage to chromosomes or plasmids [47,48]. Such a physical linkage of ARGs on the same genetic elements represents a widespread co-selection phenomenon, and it is partly through this “co-selection” mechanism that ARGs in the gut are maintained [49]. On the other hand, elevated temperature exacerbates metabolic stress, driving microbes to reduce functional competition and undergo ecological niche differentiation, thereby strengthening complementary or synergistic interactions. At the ARO level, the genetic co-localization hypothesis is strongly validated. Extremely strong positive correlations, such as *ARO:3000783* (*CmeA*, membrane fusion protein of the CmeABC multidrug efflux complex) and *ARO:3000784* (*CmeB*, inner membrane transporter of the CmeABC complex), as well as *ARO:3004032* (*tetA*(*46*), subunit of the *tetAB*(*46*) ABC transporter for tetracycline resistance) and *ARO:3004033* (*tetB*(*46*)), further support this hypothesis by ensuring the intact assembly of transport complexes, coordinated energy supply, and synchronized substrate transport. In contrast, strong negative correlations, *ARO:3002972* vs. *ARO:3004032,* likely reflect host differentiation or fitness costs linked to specific resistance mechanisms. In summary, temperature reshapes the correlation networks of ARGs by modulating gene transfer and host microbial community structure, ultimately driving the resistome toward greater integration and functional coordination under high-temperature conditions.

### 4.2. Elevated Temperature Reshapes the Diversity of CAZymes of Gut Microbiota in E. roborowskii

As ectotherms, the digestive capacity of reptiles is closely tied to the environmental temperature, which also profoundly influences the composition and function of their gut microbiota [50]. Temperature also plays a crucial role in lizard thermoregulation. When environmental temperatures exceed the lizard’s thermal optimum, resting metabolic rate increases, and additional energy must be allocated to behavioral and physiological thermoregulation to maintain body temperature [51]. Elevated energetic demand may place selective pressure on the host metabolic strategy, including reliance on microbial-assisted digestion [52]. In response, the gut microbiota may enhance the CAZyme production to improve energy harvest from dietary polysaccharides, thus compensating for the host’s increased energetic costs. These mechanisms may collectively contribute to the highest abundance of CAZymes of gut microbiota in *E. roborowskii* observed at the family level in the HT group, significantly exceeding those in the NT and ET groups. Our results showed that the HT group significantly increased the richness of CAZyme families at the family level. Although no significant differences were observed in class-level alpha diversity across treatments, family-level richness was significantly higher in the HT group. Furthermore, PCoA and PERMANOVA analyses indicated significant differences in CAZyme functional profiles among temperature treatments, suggesting that elevated temperature may enhance functional diversification related to carbohydrate metabolism by selecting specific gut microbiota.

CAZymes constitute a superfamily of enzymes responsible for the synthesis, metabolism, modification, and transport of carbohydrates. GHs include glycosidases and transglycosidases, which are responsible for the hydrolysis or transglycosylation of glycosidic bonds and play a central role in degrading complex polysaccharides such as cellulose and hemicellulose [53]. GTs are involved in the synthesis of disaccharides, oligosaccharides, and polysaccharides, as well as catalyzing the transfer of a glycosyl group from an activated donor to a specific acceptor to form glycosidic bonds and play an important role in microbial adaptation and pathogenicity [54]. CBMs are known to enhance the activity of many enzymes by targeting and promoting prolonged interactions with substrates [55]. PLs cleave uronic acid-containing polysaccharides via β-elimination, producing unsaturated enoluronic acid residues and new reducing ends [56], whereas CEs assist GHs and PLs by removing ester groups and side chains from carbohydrates [57]. AAs comprise ligninolytic enzymes and lytic polysaccharide monooxygenases, and ligninolytic enzymes exhibit synergistic effects with classical polysaccharide-depolymerizing enzymes [58]. At the class level, the combined abundance of GH, GT, and CBM is above 90%, indicating that carbohydrate metabolism mainly relies on crucial functions such as polysaccharide degradation, synthesis, and substrate binding assistance, reflecting highly efficient adaptability in carbohydrate utilization and transformation.

The relative abundance of CAZyme subfamilies is directly regulated by microbial community characteristics and indirectly regulated by environmental conditions [59]. Meanwhile, certain specialized microorganisms, such as *Asticcacaulis* and *Cytophaga,* focus on breaking down complex organic compounds, and this substrate preference can significantly influence the composition of CAZyme families in specific environments [60]. Thus, external disturbances can regulate carbohydrate metabolism by reshaping the composition of microbial communities [61]. In the NT and ET groups, *GT1*, *GT2*, and *GT4* were predominant, supporting monosaccharide transfer and the biosynthesis of structural polysaccharides and glycolipids linked to core metabolism and biomass production. Extreme niches often inhabit microbial populations with sturdy metabolic potential in harsh conditions [62]. Under high temperatures, however, a marked increase in *GT2*, *GT4*, and *CBM50* was detected, with *CBM50* becoming the third most abundant family. Elevated *GT2,* including chitin and cellulose synthases, implies enhanced synthesis of structural polysaccharides for thermal stability, while the upregulation of *GT4* may contribute to membrane stabilization through glycolipid production. Under high temperatures, however, *GT2*, *GT4*, and *CBM50* increased markedly, with *CBM50* becoming the third most abundant family. Elevated *GT2* (including chitin and cellulose synthases) implies an enhanced synthesis of structural polysaccharides for thermal stability, whereas the upregulation of *GT4* may contribute to membrane stabilization via glycolipid production. *CBM50*, which, in association with peptidoglycan or chitin hydrolysis, significantly increased, indicates an enhanced cell wall damage perception and repair ability, as well as a coordinated adaptation mechanism of microbiota. Meanwhile, the diet of *E. roborowskii,* composed of mealworms whose exoskeleton contains large amounts of chitin, might be consistent with the host’s demand for a substantial energy supply. Additionally, correlation network analysis further revealed cooperative and competitive interactions among CAZymes in response to the temperature. At the family level, positive correlations between *GT28* and *CBM50*, *GT31* and *GT48*-*GT1* imply the synergistic roles of these CAZymes not only in cell wall synthesis or modification but also in hydrolyzing chitin components in the diet to supply energy. However, negative correlations, such as *GH31* vs. *CBM50*, *GH31* vs. *GT28*, and *GH125* vs. *CBM50,* further indicate a preferential resource allocation toward cell wall synthesis and repairing rather than the hydrolysis of storage glycans, indicating an adaptive adjustment of carbon use under thermal stress.

The PPP is a glucose-oxidizing pathway that produces ribose 5-phosphate and NADPH [63]. In order to prevent damage from ROS, cells use antioxidant systems such as glutathione and thioredoxin, which rely on NADPH to regenerate reduced thiols from disulfides. In activated phagocytes, the PPP fuels this process by shifting flux away from glycolysis and generating large amounts of NADPH [64]. The increased supply of ribose-5-phosphate may support microbial growth and DNA repair under high-temperature stress by promoting nucleotide synthesis. The upregulation of *GH43* and its regulated enzymes in the HT group further verified the adaptive role of the PPP in balancing energy metabolism and stress resistance. There are significant differences in the capabilities of gut microbiota to synthesize amino acids, categorized as prototrophs (self-sufficient) and auxotrophs (exogenously dependent) [65]. Gut microbiota maintain their stability via metabolite exchange, such as shared amino acid precursors. In addition, branched-chain amino acids are not only raw materials for microbial protein synthesis but can also be absorbed into the host circulation and participate in energy metabolism [66]. Compared with the NT and ET groups, *GH3* and its regulated enzymes are upregulated in the HT group, which may play an important role in microbial protein synthesis, ensuring growth and repair under high-temperature stress, and maintaining gut microbiota stability.

### 4.3. Correlation Analysis

The gut microbiota is one of the most densely colonized microbial communities on earth, serves as an essential reservoir of ARGs, and is known as the gut resistome [67]. Previous studies have confirmed that elevated temperatures could significantly alter the composition and diversity of intestinal ARGs [24]. These changes do not occur in isolation but instead respond to temperature stress synergistically through the functional network of the microbial community. The dissemination of ARGs depends on the survival of the microorganism and the efficiency of HGT. Our study found that under high-temperature conditions, ARGs with resistance to aminoglycosides and macrolides demonstrated a significant positive correlation with the abundance of gut microbiota, which may be achieved through the enrichment of dominant microbiota. In contrast, resistance genes such as those against mupirocin exhibited a negative correlation with gut microbiota that might be due to poor adaptability to high temperatures, leading to competitive elimination. Furthermore, at the ARO level, the differential ARGs are significantly positively correlated with differential gut microbiota, which mediate diverse resistance mechanisms, including antibiotic target modification, efflux pump mechanisms, and antibiotic inactivation, suggesting that temperature exposure disrupts the compositional structure of gut microbiota, thereby influencing the resistome. Additionally, another possible reason is that gut microbiota gain a competitive advantage by synthesizing antibiotics to inhibit the growth of other microorganisms, which may also lead to the maintenance and spread of ARGs.

In addition to regulating intestinal pH to maintain microenvironmental stability, *Tetragenococcus* is also known to indirectly support host energy metabolism through metabolites, including lactic acid [68]. *Faecalicatena* exerts protective effects by interactions with both the intestinal microenvironment and the host and may also participate in propionic acid production [69]. Under high-temperature stress, host metabolism is accelerated, which leads to an increase in energy consumption and impairs the intestinal barrier function. Therefore, we speculate that *E. roborowskii* may enrich these two bacteria to enhance energy metabolism and utilize short-chain fatty acids (SCFAs) for barrier repair to cope with rising temperatures. CAZymes represent microorganisms’ adaptive strategies for carbon source utilization. Previous studies have indicated that GHs play a crucial role in polysaccharide degradation [70].

Specifically, β-glucosidases (BGLs, EC 3.2.1.21) within the *GH3* family are recognized as key enzymes in lignocellulose hydrolysis and biofuel production, which could convert cellobiose and other cellooligosaccharides generated by cellulases into fermentable sugars [71]. Endo-β-1,4-galactanases, belonging to the *GH53* family, degrade β-1,4-linked galactans and arabinogalactans [72]. *GH43* is considered one of the largest reservoirs of xylosidases, together with families *GH3*, *GH39*, and *GH52*. Additionally, *CBM74* family proteins specifically bind to granular starch, enhancing the capacity of α-amylases to hydrolyze resistant starches (RSs) [73]. In this study, positive correlations were observed between *GH3, GH53*, and *GH43* and genera such as *Tetragenococcus* and *Faecalicatena*, indicating that these taxa may contribute to enhancing polysaccharide degradation for the sake of host energy demands under high temperatures. Conversely, GT and AA classes exhibited negative correlations. GTs contribute to glycan synthesis and microbial adaptation, whereas AAs are responsible for the modification of complex carbon sources. Families such as *GT31*, *PL4*, and *CBM47* were also negatively correlated, suggesting gut microbiota may downregulate costly energy processes in favor of stress response and more efficient energy acquisition under heat stress.

Carbohydrate metabolism consists of the utilization of glucose and monosaccharides through intermediary pathways, including the citric acid cycle [74]. It has been shown that several cells are able to resist heat stress damage and maintain protein synthesis by enhancing intracellular amino acid absorption and metabolism [75]. At the secondary level, pathways including carbohydrate metabolism, membrane transport, and amino acid metabolism were significantly positively correlated with *GH53*, *GH3*, and *GH43*, which may be attributed to high-temperature conditions necessitating greater energy metabolism. Meanwhile, amino acid metabolism may contribute to the maintenance of protein homeostasis by supplying precursors. Membrane transport pathways, such as ABC transporters, may enhance nutrient uptake efficiency and facilitate the acquisition of degraded carbohydrate fragments and amino acids [76]. Notably, elevated temperature increases membrane fluidity and permeability when coupled with upregulated membrane transport proteins, including the synergistically expressed *tetA*(*46*) and *tetB*(*46*) of the tetracycline resistance transporter, which may create favorable conditions for the horizontal transfer of ARGs. At the tertiary level, GHs exhibited positive correlations with carbohydrate metabolism, the PPP, amino acid metabolism, lipid metabolism, and antibiotic biosynthesis, suggesting that gut microbiota may enhance polysaccharide degradation to supplement energy reserves and metabolic precursors, thereby achieving a rapid adaptation to stress conditions. Additionally, CAZymes and KEGG metabolic pathways have also been associated with diet [11]. In our study, lipid metabolism may be linked to the dietary intake of large quantities of mealworms, which serve as a lipid-rich nutrient source. Under stress conditions, gut microbiota can secrete antibacterial compounds to enhance their colonization ability. Therefore, a significant positive correlation between GHs and antibiotic biosynthesis pathways may be beneficial for their niche competition and heat stress defense. Collectively, under high-temperature stress, gut microbiota in *E. roborowskii* regulate CAZymes to enhance carbon utilization, redistributing metabolic resources to prioritize balanced energy metabolism.

## 5. Conclusions

**Figure 9:**
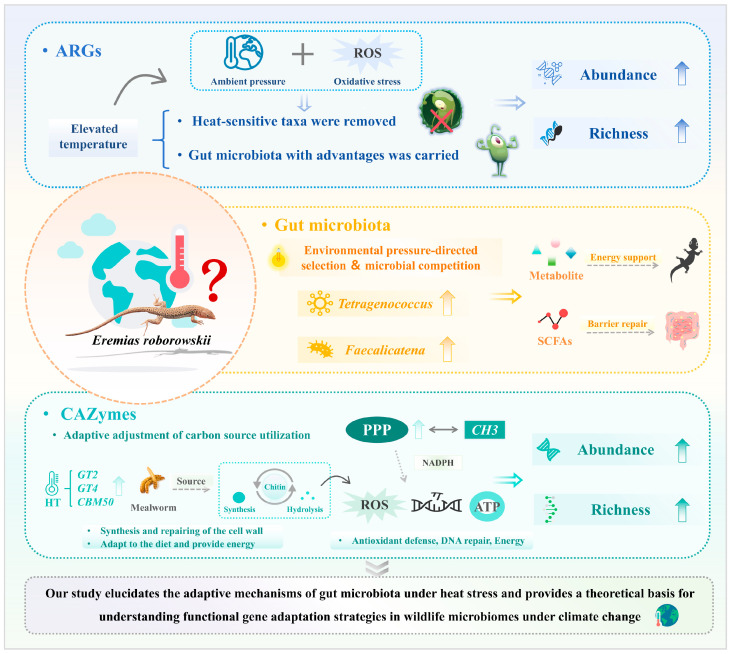
The response mechanism of gut microbiota and functional genes in *E. roborowskii* under heat stress.

Our study demonstrates that elevated temperatures may influence the gut microbiota through the enrichment of heat-tolerant microbial taxa, alteration of the transfer efficiency of ARGs, and optimization of the metabolic allocation of CAZymes, to exert significant effects on the gut microbiota function in *E. roborowskii*. As shown in Figure 9, under heat stress, the gut microbiota of *E. roborowskii* was enriched in *Tetragenococcus* and *Faecalicatena*, which produce metabolites for energy metabolism and SCFAs that help to maintain the intestinal barrier. Furthermore, both the abundance and richness of ARGs and CAZymes were found to increase. On the one hand, elevated temperature caused ambient pressure and oxidative stress that altered the profile of ARGs by removing heat-sensitive taxa and enriching the heat-tolerant, fitness-enhancing taxa. On the other hand, elevated temperature induced metabolic remodeling, leading to the upregulation of CAZymes such as *GT2*, *GT4*, and *CBM50*, which further indicated a preferential allocation of resources toward cell wall synthesis and repair. This adaptive adjustment in carbon utilization corresponds to the host’s diet of chitin-rich mealworms, meeting the substantial energy demands of *E. roborowskii* under high-temperature conditions. The upregulation of the *GH3* family and the PPP provides critical evidence for microbial antioxidant defense, growth, and repair. In summary, these changes may lead to a gut microbiota imbalance, a mismatch between host energy supply and demand, and an increased risk of inflammation, which may ultimately result in a contraction of the species’ niche and a reduction in adaptability. Our study provides critical insights into the response patterns and functional adaptations of ARGs and CAZymes in the gut microbiota of reptiles under global warming, offering a scientific basis for targeted conservation strategies for desert lizards, particularly *E. roborowskii*, as well as theoretical support for biodiversity conservation and the maintenance of ecosystem stability in the arid desert regions of Xinjiang.

## Data Availability

The data supporting the findings of this study are publicly available from the National Center for Biotechnology Information (NCBI) Sequence Read Archive (SRA) at https://www.ncbi.nlm.nih.gov/sra/PRJNA1434117 (accessed on 9 March 2026), reference number [PRJNA1434117]. These data are currently under restricted access, but the data can be accessed via the following temporary link: https://dataview.ncbi.nlm.nih.gov/object/PRJNA1434117?reviewer=4dnhdig3na5t04bbmgnq3jm01o (accessed on 9 March 2026).

## Supplementary Materials

The following supporting information can be downloaded at https://www.mdpi.com/article/10.3390/microorganisms14051084/s1. Figure S1: Clean read base distribution in *E. roborowskii* under different temperature treatment; Figure S2: Clean read base quality distribution in *E. roborowskii* under different temperature treatment.
