# Supplementary material for: Temperature Elevation Alters the Gut Antibiotic Resistome and Carbohydrate-Active Enzymes in the Desert Lizard Eremias roborowskii
Source: Microorganisms. 2026 May 11;14(5):1084. doi: 10.3390/microorganisms14051084 (PMC13209948; doi:10.3390/microorganisms14051084)
Supplement: Supplementary file 1 [file microorganisms-14-01084-s001.zip › microorganisms-4247865-supplementary.pdf]

## Supplementary Information (SI)

# Temperature elevation alters the gut antibiotic resistome and carbohydrate-active enzymes in the desert lizard *Eremias roborowskii*

## Supplementary Figures

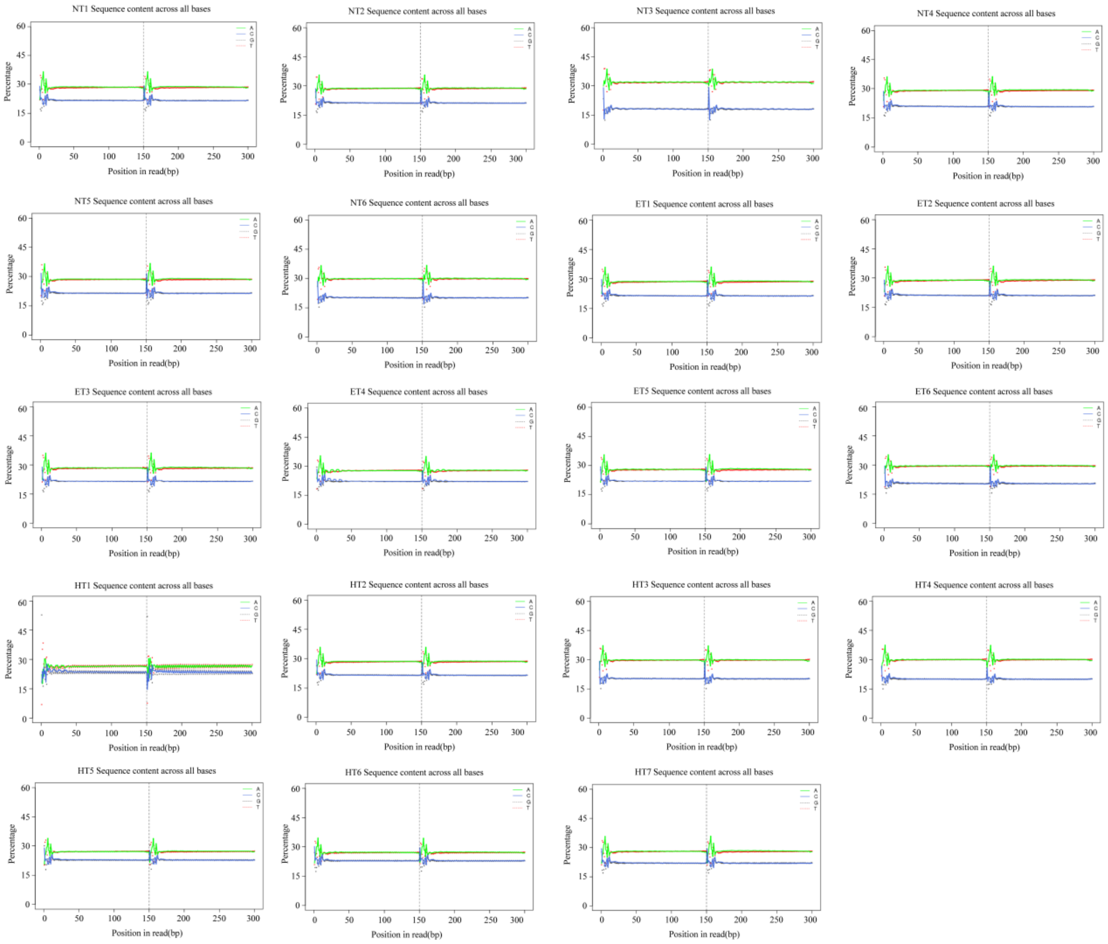


**Supplementary Figure S1. Clean read base distribution in *E. roborowskii* under different temperature treatment.**

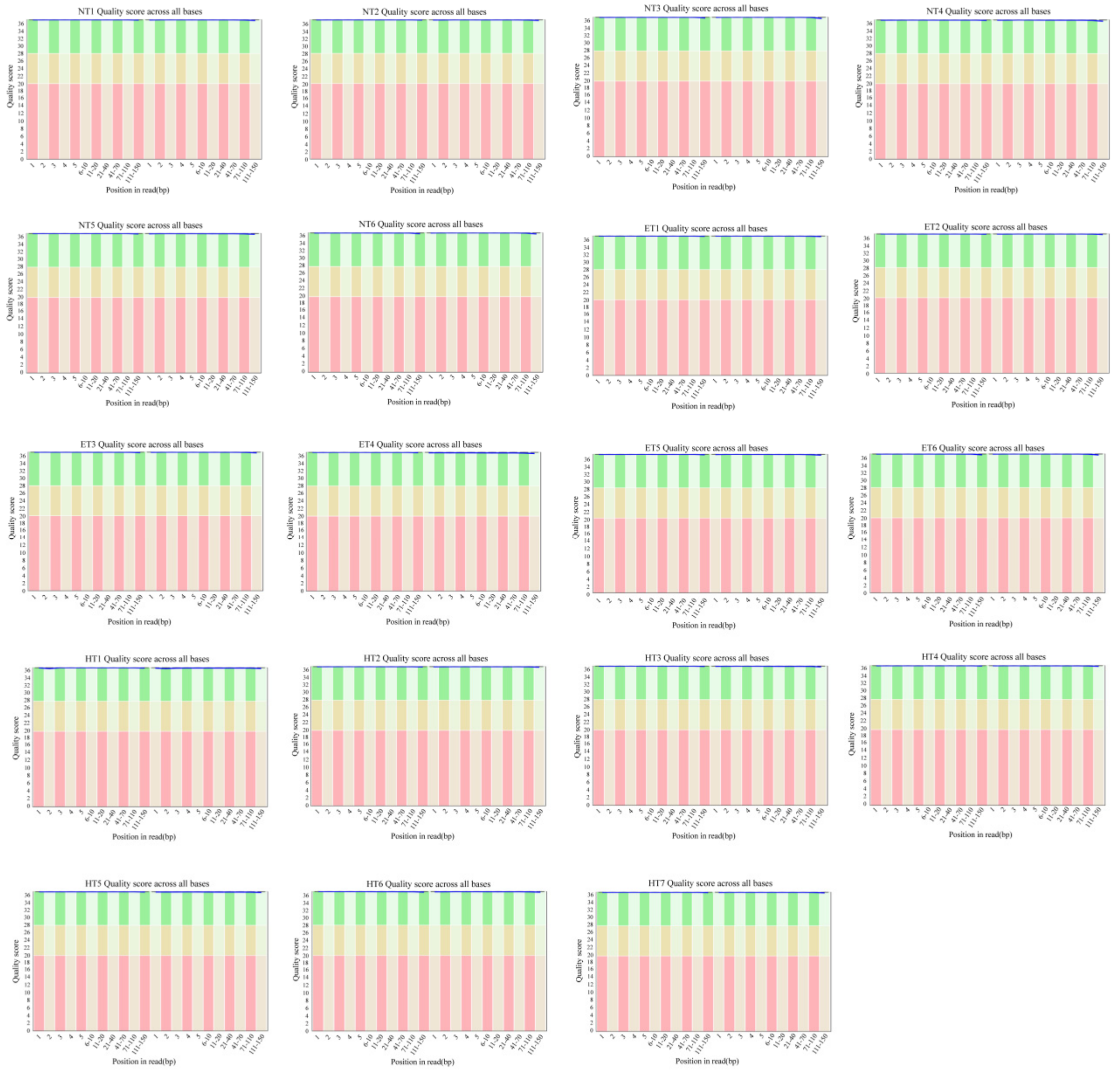


**Supplementary Figure S2. Clean read base quality distribution in *E. roborowskii* under different temperature treatment.**
